# Reasons for lithium discontinuation in men and women with bipolar disorder: a retrospective cohort study

**DOI:** 10.1186/s12888-018-1622-1

**Published:** 2018-02-07

**Authors:** Louise Öhlund, Michael Ott, Sofia Oja, Malin Bergqvist, Robert Lundqvist, Mikael Sandlund, Ellinor Salander Renberg, Ursula Werneke

**Affiliations:** 1 Sunderby Research Unit, Department of Clinical Sciences, Division of Psychiatry, Umeå University, Umeå, Sweden; 2 Department of Public Health and Clinical Medicine, Division of Medicine, Umeå University, Umeå, Sweden; 3 Department of Psychiatry, Sunderby Hospital, Luleå, Sweden; 4 Department of Psychiatry, Piteå Älvdals Hospital, Piteå, Sweden; 5 Research and Innovation Unit, Luleå, Norrbotten Region Sweden; 6 Department of Clinical Science, Division of Psychiatry, Umeå University, Umeå, Sweden; 7 Sunderby Hospital – Psychiatry, 97180 Luleå, Sweden

**Keywords:** Lithium, Bipolar disorder, Physical health, Compliance, Side effects

## Abstract

**Background:**

Lithium remains first choice as maintenance treatment for bipolar affective disorder. Yet, about half of all individuals may stop their treatment at some point, despite lithium’s proven benefits concerning the prevention of severe affective episodes and suicide.

**Methods:**

Retrospective cohort study in the Swedish region of Norrbotten into the causes of lithium discontinuation. The study was set up to (1) test whether patients with bipolar affective disorder or schizoaffective disorder, treated with lithium maintenance therapy, were more likely to discontinue lithium because of adverse effects than lack of therapeutic effectiveness, (2) explore gender differences, (3) understand the role of diagnosis and (4) identify who, patient or doctor, took the initiative to stop lithium. Review of medical records for all episodes of lithium discontinuation that had occurred between 1997 and 2013 with the intent to stop lithium for good.

**Results:**

Of 873 patients treated with lithium, 54% discontinued lithium, corresponding to 561 episodes of lithium discontinuation. In 62% of episodes, lithium was discontinued due to adverse effects, in 44% due to psychiatric reasons, and in 12% due to physical reasons interfering with lithium treatment. The five single most common adverse effects leading to lithium discontinuation were diarrhoea (13%), tremor (11%), polyuria/polydipsia/diabetes insipidus (9%), creatinine increase (9%) and weight gain (7%). Women were as likely as men to take the initiative to stop lithium, but twice as likely to consult a doctor before taking action (*p* < 0.01). Patients with type 1 BPAD or SZD were more likely to discontinue lithium than patients with type 2 or unspecified BPAD (*p* < 0.01). Patients with type 1 BPAD or SZD were more likely to refuse medication (*p* < 0.01). Conversely, patients with type 2 or unspecified BPAD were three times as likely to discontinue lithium for lack or perceived lack of effectiveness (*p* < 0.001).

**Conclusions:**

Stopping lithium treatment is common and occurs mostly due to adverse effects**.** It is important to discuss potential adverse effects with patients before initiation and continuously during lithium treatment, to reduce the frequency of potentially unnecessary discontinuations.

## Background

Ever since publication of the BALANCE trial in 2010 [1], lithium has experienced a renaissance as maintenance treatment for bipolar affective disorder (BPAD) in Europe. In 2014, the UK National Institute for Clinical Excellence (NICE) endorsed lithium as a first-line, long-term pharmacological treatment for bipolar disorder [2]. Two recent large observational register studies have demonstrated lithium’s therapeutic superiority regarding prevention of suicide and recurrence of acute affective episodes [3, 4].

Yet, despite its therapeutic superiority, patients may find it difficult to take lithium long-term. Non-adherence is common in the treatment of serious mental disorders, irrespective of the substance in question [5]. For lithium, non-adherence rates vary from 14% to 61% [6, 7]. Both doctors and patients may discontinue lithium, albeit for different reasons [8]. But as lithium may remain the treatment of choice for most patients requiring maintenance treatment even in the face of adverse renal effects [9, 10], it is important to understand the reasons for discontinuing lithium. Equally important is it to understand the factors that influence the initiative to stop.

### Aim

We conducted a retrospective historical cohort study to (1) test whether patients with BPAD or schizoaffective disorder (SZD), treated with lithium maintenance therapy, were more likely to discontinue lithium because of adverse effects than lack of therapeutic effectiveness, (2) explore gender differences, (3) understand the role of diagnosis and (4) identify who, patient or doctor, took the initiative to stop lithium.

## Method

### Study design

We designed a retrospective (historical) cohort study (LISIE) into effects and side effects of lithium treatment as compared to other mood stabilizers for the maintenance treatment of BPAD. The Regional Ethics Review Board at Umeå University, Sweden, had approved this study (DNR 2010-227-31 M, DNR 2011-228-32 M, DNR 2014-10-32 M).

The participants were informed about the nature of the study in writing and provided verbal informed consent. The consent was documented in our research files, dated and signed by the research worker who obtained the consent. In accordance with the ethics approval granted, for deceased patients, no consent was obtained. The ethics committee approved these consent procedures prior to commencement of this study.

### Participants

For LISIE, we identified all patients in the Swedish region of Norrbotten, who had been either diagnosed with BPAD (F31) or SZD (F25), or who had been prescribed lithium and were at least 18 years of age. We screened the medical records of all patients who had either given informed consent to participate, or who we were approved to include because they had deceased.

For this study, we retrospectively examined routine clinical data recorded until 31 December 2015. The extraction, validation and analysis were performed in 2016 and 2017. We defined as “exposed”, patients who had received a diagnosis of BPAD or SZD on at least two occasions at least six months apart any time between 1997 and 2013, and for whom at least two positive lithium serum levels were available. We determined when lithium had been started and validated the diagnoses in the medical records at this point.

Patients were potentially eligible for inclusion into this study, when we found a lithium prescription that had been discontinued in the electronic prescription database. We included all episodes for which the medical records then indicated that lithium was indeed discontinued with the intention to stop for good. At this point, we also checked the records for the exact point of time lithium was discontinued, since this may have preceded discontinuation of the prescription.

### Chart review, variable definitions and outcomes

For each patient, we systematically abstracted the case records to obtain information regarding (1) baseline characteristics including diagnosis, (2) date of and reason for lithium discontinuation and (3) communication about the initiative to stop lithium. All episodes of lithium discontinuation were registered in the time frame studied. We used this information to quantify overall reasons for lithium discontinuation. We also explored for how long a patient had coped with a side effect or a doctor had accepted an adverse effect before discontinuing lithium.

We stratified diagnosis in two groups, BPAD type 1 or SZD on the one hand and BPAD type 2 or unspecified on the other. We divided reasons for lithium discontinuation into three main categories. The first category concerned psychiatric reasons, related to the mental disorder itself or associated circumstances, such as not being able to pay for medication. The second category related to physical health reasons that could interfere with lithium. The third category covered adverse effects. We allocated intentional overdoses to the first category of psychiatric reasons. We allocated unintentional overdoses and unintended increases in lithium serum concentration to the second category of physical health reasons, such as chronic kidney disease, dehydration or co-medications interfering with lithium pharmacokinetics. For the psychiatric reason category, we created a variable called “non-adherence”. Under this variable, we summed up discontinuation of lithium due to fear of adverse effects, being in disagreement with the diagnosis, refusing medication, feeling subjectively well and not adhering to monitoring.

We determined who initiated lithium discontinuation, patient or doctor. This, we called “agent who took the initiative to discontinue lithium”. For the decision process, we explored whether patients had consulted their doctor before. Some patients had discontinued lithium only once in the time frame of our study. But others had discontinued and then reinstated lithium on at least one occasion. Thus, we divided episodes of discontinuation into first and subsequent episodes.

We conducted a sub-analysis, to assess how long a patient had coped with or a doctor had accepted an adverse effect before discontinuing lithium. To avoid overestimation of the length of time a problem had persisted before it led to discontinuation of lithium (lead time), we only considered *proven* first but not subsequent episodes of lithium discontinuation. For this sub-analysis, we traced back medical records until 1965 to make sure that we had not missed any previous episodes of lithium discontinuation, which might have occurred prior to 1997. Here, we excluded patients for whom such prior episodes of lithium discontinuation were found or where information was missing.

### Control for bias

Of all patients approached, 75% consented to inclusion into the LISIE study. In accordance with the ethical approval granted, we controlled for selection bias in the whole LISIE study, comparing age, sex, maximum recorded lithium and creatinine level as key parameters, available in anonymized form. We did not find any significant difference between participating and non-participating patients. To minimize observer and recording bias, all reasons for discontinuation were assessed by two separate reviewers. Patients, who were excluded from the final sample since the reasons for stopping lithium had not been recorded, had the same age and sex distribution as patients who were included.

#### Sub-analysis lead time

In a sub-analysis, we explored the lead time from start of lithium treatment until the first episode of discontinuation. We included only patients for whom we could unequivocally define the first episode of lithium discontinuation. Age and sex were not significantly different in patients included and excluded from this sub-analysis.

### Statistical analysis

We conducted a descriptive analysis, establishing the frequency of all variables in our database. We divided the sample according to category of reason, and stratified then further according to gender, diagnosis and agent, patient or doctor, who took the initiative to discontinue lithium. To assess potential group differences, we used chi square test for categorical variables and t-test and ANOVA for continuous variables. Statistical significance level was set at *p* ≤ 0.05. We conducted the statistical analysis with IBM SPSS Statistics (Version 23). We checked our whole method against the Strobe checklist (Appendix 1).

## Results

### Baseline characteristics

**Fig. 1:**
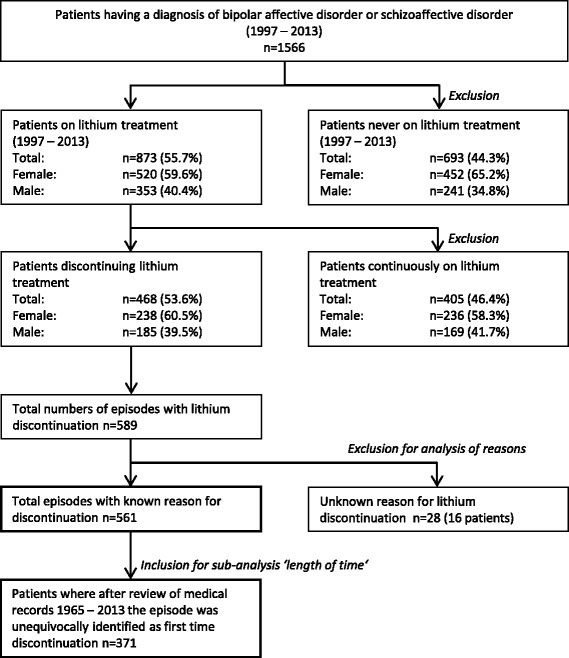
Selection of study sample

The total cohort comprised of 1566 patients. Of these, 873 had received lithium at some point during the study period. 468 (54%) patients discontinued lithium at least on one occasion with intention to stop for good. Of these, 81% of patients had stopped lithium on one occasion and 19% on more than one occasion after at least one futile attempt at lithium reinstatement. In total, there were 589 episodes of lithium discontinuation. For 561 (95%) episodes, the reasons of lithium discontinuation were known (Fig. 1). For these episodes, 922 individual reasons were recorded (Table 1).

**Table 1:** Baseline characteristics of the sample of patients having discontinued lithium treatment at least once between the years of 1997-2013

Gender, n (%)	
Female	238 (60.5)
Male	185 (39.5)
Type of disorder, n (%)	
BPAD 1/SZD	199 (42.5)
BPAD 2/Other	269 (57.5)
Total number of episodes with lithium discontinuation, n (%)	589
Patients with one episode only	378 (80.8)
Patients with more than one episode	90 (19.2)
Total number of episodes with known reason for discontinuation, n (%)	561
Episodes with one reason only	355 (63.3)
Episodes with more than one reason	206 (35.7)
Total number of reasons for lithium discontinuation, n (%)	922
Patients where first episode of discontinuation where identified, n (%)	371 (79.3)
Lead-time to first lithium discontinuation, years	
Mean (SD)	3.6 (6.1)
Median (min-max)	1.1 (0.02-39.9)

*n* number, *BPAD* bipolar affective disorder, *SZD* schizoaffective disorder, *Other* unspecified bipolar affective disorder or subgroup specified otherwise; *SD* standard deviation

### Reasons for lithium discontinuation

Adverse effects dominated reasons for lithium discontinuation with 62% of the 561 episodes. Psychiatric reasons were given in 44% and physical health reasons in 12% of all episodes (*p* < 0.001) (Table 2). In 37% of episodes, there were more than one reason for lithium discontinuation reported. As there were more reasons than episodes, the percentages did not add up to 100.

**Table 2:** Reasons for lithium discontinuation

	Total, n (%)	Type of disorder, n (%)		Agent, n^a^ (%)	
		BPAD 1/SZD	BPAD2/ Other	Doctor	Patient
Episodes, n	561	253	308	225	334
Psychiatric reasons					
Total	249 (44.4)	97 (38.3)	152 (49.4)**	81 (36.0)	167 (50.0)**
Non-adherence	121 (21.6)	65 (25.7)	56 (18.2)*	26 (11.6)	95 (28.4)***
Fear for adverse effects	17 (3.0)	6 (2.4)	11 (3.6)	2 (0.9)	15 (4.5)*
Not agreeing with diagnosis	6 (1.1)	5 (2.0)	1 (0.3)	0 (0)	6 (1.8)*
Refusing medication	34 (6.1)	23 (9.1)	11 (3.6)**	0 (0)	34 (10.2)***
Feeling subjectively well	26 (4.6)	12 (4.7)	14 (4.5)	0 (0)	26 (7.8)***
Not adhering to monitoring	38 (6.8)	19 (7.5)	19 (6.2)	24 (10.7)	14 (4.2)**
Perceived or actual lack of effectiveness	116 (20.7)	25 (9.9)	91 (29.5)***	46 (20.4)	69 (20.7)
Intentional Li intoxication	5 (0.9)	0 (0)	5 (1.6)*	5 (2.2)	0 (0)**
Other reasons^b^	22 (3.9)	13 (5.1)	9 (2.9)	11 (4.9)	11 (3.3)
Physical health reasons					
Total	68 (12.1)	36 (14.2)	31 (10.1)	53 (23.6)	17 (4.2)***
Unintentional Li intoxication	11 (2.0)	6 (2.4)	5 (1.6)	10 (4.4)	1 (0.3)**
Increase of Li concentration	30 (5.3)	19 (7.5)	11 (3.6)*	28 (12.4)	2 (0.6)***
Pregnancy or planned pregnancy	15 (2.7)	5 (2.0)	10 (3.2)	5 (2.2)	10 (3.0)
Physical health problem^c^	12 (2.1)	6 (2.4)	6 (1.9)	11 (4.9)	1 (0.3)***
Adverse effects					
Total	350 (62.4)	164 (64.8)	186 (60.4)	142 (63.1)	207 (62.0)
Kidneys and urinary tract					
Creatinine increase, Li-nephropathy	51 (9.1)	37 (14.6)	14 (4.5)***	44 (19.6)	7 (2.1)***
Nephrogenic DI, polyuria, polydipsia	52 (9.3)	27 (10.7)	25 (8.1)	25 (11.1)	27 (8.1)
Endocrine system					
TSH increase, hypothyroidism	11 (2.0)	4 (1.6)	7 (2.3)	6 (2.7)	5 (1.5)
CNS					
Tremor	61 (10.9)	32 (12.6)	29 (9.4)	27 (12.0)	34 (10.2)
Dizziness	12 (2.1)	4 (1.6)	8 (2.6)	6 (2.7)	6 (1.8)
Headache	8 (1.4)	1 (0.4)	7 (2.3)	2 (0.9)	6 (1.8)
Restless legs	5 (0.9)	2 (0.8)	3 (1.0)	2 (0.9)	3 (0.9)
Psychological adverse effects					
Emotional blunting	31 (5.5)	9 (3.6)	22 (7.1)	2 (0.9)	29 (8.7)***
Irritability/anxiety	12 (2.1)	2 (0.8)	10 (3.2)*	4 (1.8)	8 (2.4)
Other, not specified	20 (3.6)	7 (2.8)	13 (4.2)	5 (2.2)	15 (4.5)
Cognitive adverse effects					
Fatigue	30 (5.3)	11 (4.3)	19 (6.2)	13 (5.8)	17 (5.1)
Imoaired concentration	17 (3.0)	3 (1.2)	14 (4.5)*	11 (4.9)	6 (1.8)*
Impaired memory	8 (1.4)	1 (0.4)	7 (2.3)	6 (2.7)	2 (0.6)*
Gastrointestinal tract					
Diarrhoea	71 (12.7)	33 (13.0)	38 (12.3)	21 (9.3)	50 (15.0)*
Nausea	24 (4.3)	6 (2.4)	18 (5.8)*	7 (3.1)	17 (5.1)
Stomach ache	10 (1.8)	4 (1.6)	6 (1.9)	0 (0)	10 (3.0)**
Other, not specified	7 (1.2)	2 (0.8)	5 (1.6)	2 (0.8)	5 (1.6)
Skin					
Psoriasis	10 (1.8)	6 (2.4)	4 (1.3)	6 (2.7)	4 (1.2)
Acne	8 (1.4)	4 (1.6)	4 (1.3)	3 (1.3)	5 (1.5)
Other					
Weight gain	41 (7.3)	19 (7.5)	22 (7.1)	12 (5.3)	29 (8.7)
Edema	19 (3.4)	10 (4.0)	9 (2.9)	8 (3.6)	10 (3.0)
Muscle weakness	13 (2.3)	6 (2.4)	7 (2.3)	7 (3.1)	6 (1.8)
Xerostomia	5 (0.9)	2 (0.8)	3 (1.0)	1 (0.4)	4 (1.2)
Other adverse effects^b^	64 (11.4)	29 (11.5)	35 (11.4)	27 (12.0)	37 (11.1)

*n* number, *BPAD* bipolar affective disorder, *SZD* schizoaffective disorder, *Other* unspecified or otherwise specified bipolar affective disorder, *DI* diabetes insipidus

^a^*n* = 559, data missing in 2 (0.4%) episodes; ^b^frequency less than five episodes; ^c^i.e. comorbidities as dementia, chronic heart failure, cancer, non-lithium related chronic kidney disease, *p < 0.05, **p < 0.01, ***p < 0.001

Concerning psychiatric reasons, non-adherence was reported in 22% of episodes and perceived or actual lack of effectiveness in 21% of episodes. Of all patients who had discontinued lithium due to perceived or actual lack of effectiveness, 26% reinstated lithium subsequently. The five single most common adverse effects leading to lithium discontinuation included diarrhoea (13%), tremor (11%), polyuria/polydipsia/diabetes insipidus (9%), creatinine increase (9%) and weight gain (7%). Hyperparathyroidism, hypernatraemia and sexual dysfunction were only rarely given as reasons for discontinuation and accounted for less than five episodes each. Intentional overdoses were rarely a cause for discontinuing lithium for good. Unintentional overdoses and risk of increasing lithium concentration accounted for 7% of all discontinuation episodes.

#### Gender differences

Men with BPAD or SZD were more often treated with lithium (59%) than women (54%, *p* < 0.05). There was no significant difference in proportion of women and men who had continued lithium compared to those who had discontinued lithium. But women had three times more often discontinued lithium because of weight gain (*p* < 0.01) and five times as often because of oedema (*p* < 0.01). In 4 % of episodes, women discontinued lithium due to pregnancy. Conversely, men had three times more often stopped lithium for feeling well (*p* < 0.01). Men had twice as often discontinued lithium without consulting a doctor (*p* < 0.01).

#### Type of disorder

Patients with type 1 BPAD or SZD were more likely to discontinue lithium than patients with type 2 or unspecified BPAD (*p* < 0.01). Patients with type 1 BPAD or SZD were more likely to refuse medication (*p* < 0.01). Conversely, patients with type 2 or unspecified BPAD were three times as likely to discontinue lithium for lack or perceived lack of effectiveness (*p* < 0.001). Creatinine increases accounted for three times as many episodes of lithium discontinuation in patients with type 1 BPAD or SZD (*p* < 0.001). Unintentional increase of lithium concentration accounted for twice as many episodes of lithium discontinuation in patients with type 1 BPAD or SZD (*p* < 0.05) (Table 2).

#### Agent who took the initiative to discontinue lithium

Patients were more likely to take the initiative to stop lithium than doctors (*p* < 0.001). Of all discontinuation episodes, 28% were attributable to lack of adherence on the part of the patient (*p* < 0.001). Concerning adverse effects, patients discontinued lithium more often than doctors because of emotional blunting (*p* < 0.001), diarrhoea (*p* < 0.05) and stomach ache (*p* < 0.01). Doctors discontinued prescribing lithium if patients had not stuck to the required follow-up (*p* < 0.01). Doctors also stopped prescribing lithium for fear of intoxications, irrespective of whether such were intentional or not (*p* < 0.01). Finally, doctors stopped lithium more commonly due to creatinine increases and chronic kidney disease (p < 0.001).

### Top 10 reasons for lithium discontinuation in relation to duration of treatment

This sub-analysis was based on the first episode of lithium discontinuation in those 371 patients for whom we could be sure that this was unequivocally their first episode of lithium discontinuation. In this subgroup, patients had taken lithium for a mean of 3.6 years (SD 6.1) before stopping treatment. 50% of patients discontinued within 1.1 years (min 6 days, max 39.9 years). Judged on the duration of lithium treatment before discontinuation, patients with type 1 BPAD or SZD coped with adverse effects for significantly longer periods of times than patients with type 2 or unspecified BPAD (*p* < 0.01). Nausea and diarrhoea tended to lead to early discontinuation of lithium. Nausea accounted for discontinuation of lithium in 21 episodes and diarrhoea in 49 episodes (Table 3). Nausea was tolerated for half a year on average. The longest time nausea was tolerated for was one year and seven months. Diarrhoea was tolerated for one year and seven months on average. Yet, a substantial number of patients tried to cope with diarrhoea for a much longer time. There were thirteen patients who took lithium for at least two years before finally stopping due to diarrhoea. Renal factors tended to lead to lithium discontinuations later. Polyuria, polydipsia and diabetes insipidus led to lithium discontinuation usually within five years. Lithium discontinuation due to rising creatinine level occurred on average after 17 years. Four patients discontinued lithium after 30 years due to increased creatinine.

**Table 3:** Top 10 reasons for lithium discontinuation in relation to duration to lithium treatment (*n* = 371)^a^

			Lead time to lithium discontinuation	
		n (%)	Years, mean (SD)	Years, median (min-max)
1.	Perceived or actual lack of effectiveness	90 (24.6)	2.3 (4.9)	1.1 (0.04-39.9)
2.	Non-adherence	85 (22.4)	2.9 (3.6)	1.2 (0.1-15.0)
3.	Diarrhoea	49 (13.2)	1.6 (2.2)	0.7 (0.02-10.5)
4.	Tremor	45 (12.1)	3.7 (5.4)	1.6 (0.02-23.3)
5.	Weight gain	35 (9.4)	2.6 (3.3)	1.3 (0.1-12.9)
6.	Nephrogenic diabetes insipidus, polyuria, polydipsia	32 (8.6)	4.8 (5.5)	2.7 (0.1-20.6)
7.	Emotional blunting	24 (6.5)	2.2 (1.9)	1.8 (0.1-6.4)
8.	Fatigue	22 (5.9)	2.6 (4.8)	0.4 (0.02-17.3)
9.	Nausea	21 (5.7)	0.5 (0.5)	0.3 (0.02-1.6)
10.	Creatinine increase, Li-nephropathy	20 (5.4)	17.2 (11.2)	17.0 (0.1-38.8)

*n* number, *SD* standard deviation

^a^Sub-analysis of 371 patients

## Discussion

In our study, 54% of patients treated with lithium discontinued their medication on at least one occasion with the intention to stop for good. This is well in the range of estimates reported (6). This is the first study that systematically identifies reasons for discontinuation of lithium in a large sample of patients, over a sufficiently long follow-up period to take into account adverse effects occurring late during the course of the illness. We found four more studies exploring reasons for lithium discontinuation. These stem from the 1980s and 90s, with sample sizes ranging from 20 to 64 patients and follow-up times from 6 months to 7 years (11-14).

### Usual suspects?

Adverse effects were the most common cause for lithium discontinuation. Among the adverse effects, diarrhoea, tremor, creatinine increase, polyuria/polydipsia/diabetes insipidus and weight gain were the top five reasons for discontinuing lithium. This is in line with findings of three of the previous studies, which also found that adverse event was the most common reason for lithium discontinuation [11–13]. However, Schumann et al. found that lithium discontinuation occurred more commonly due to “internal resistance to treatment” than adverse effects [14]. Yet, due to the relative short follow-up times, neither of the previous studies were designed to capture concerns about impairment of glomerular function. This kind of problem tends to appear only after decades of treatment [15, 16]. Only very few patients had their lithium stopped because of hypercalcaemia/hyperparathyroidism, an adverse effect classically associated with lithium [17].

Psychiatric reasons were given in 44% of all episodes. This calls for further developing of strategies to improve adherence. One fourth of patients who had discontinued lithium due to actual or perceived lack of effectiveness had to reinstate lithium subsequently. Better identification of subgroups of patients who have the most to gain from lithium treatment may help to reduce the amount of lithium discontinuation leading to recurrence of affective episodes.

### Gender differences

Men and women were equally likely to discontinue lithium. However, men and women discontinued for different reasons. More men stopped because they felt well. Also, they were less likely to speak to a doctor before ceasing medication. This suggests that clinicians need to engage more actively with men. Subjective well-being, although being desirable, may increase the risk of treatment discontinuation. Strategies, such as shared decision-making (SDM) [18, 19], motivational interviewing, psychoeducation and involvement of relatives [7, 20], dedicated mood disorder clinics [7], taking account of executive function in older patients [21], or offering financial incentives for patients with psychotic disorders [22], may improve adherence. However, we do not know how well such strategies work specifically in the context of lithium treatment. Neither do we know how far such strategies can help patients to cope with adverse effects of their treatment.

Women were more likely to stop lithium for fear of weight gain**.** Women may be more susceptible to changes in body image and pressures of social norms that can make lithium mediated weight gain unacceptable [23, 24]. This finding is in line with a study by Kriegshauser et al. 2010 [25]. In that study, men and women had the same risk of weight gain. Yet, women were twice as likely to regard weight gain as the worst of all adverse effects. Interventions could target the underlying aetiology. For instance, lithium associated weight gain could be secondary to hypothyroidism. This underlines the importance of regular thyroid function tests. Polyuria, leading to polydipsia, leading to an increased intake of high caloric sugary drinks is another potential cause. When thinking diet, patients and doctors alike may focus on foods rather than drinks. Yet, sugar-sweetened drinks, such as soft drinks *and* juices, are a major contributor to obesity world-wide [26]. Finally, lithium may also have direct weight increasing properties. As demonstrated in animal experiments, lithium can increase the expression of melanin concentrating hormone (MCH), a powerful orexic peptide [27]. Women were also more likely to discontinue lithium because of oedema. Such may occur due to higher oestrogen levels in women, which could promote water retention in body tissues [28].

### Type of disorder

Patients with type 1 BPAD or SZD were more likely not to accept lithium treatment in the first place, whereas patients with type 2 or unspecified BPAD discontinued lithium treatment due to lack of perceived effectiveness. This calls for a further evaluation of the role of lithium maintenance treatment for type 2 BPAD. Concerns about renal function and creatinine increases led more frequently to lithium discontinuation in patients with type 1 BPAD or SZD. Patients with type 2 or unspecified BPAD may be more likely to discontinue earlier because they seem to have less therapeutic benefit. Thus, they may be less likely to accrue sufficiently long lithium exposure for clinically significant glomerular impairment to develop.

### Agent who took the initiative to discontinue lithium

Unsurprisingly, patients were more likely to discontinue lithium when lacking insight or not wanting to take medication. Significantly more patients with type 1 BPAD or SZD discontinued lithium. This may reflect a greater loss of function leading to lower adherence. Equally, doctors may be more likely to prescribe lithium to patients with type 1 BPAD or SZD. In this patient group, doctors may be particularly concerned about the risk of recurrence and suicide and hence more likely to disregard patients’ concerns about adverse effects. They may also be less likely to inform these patients about potential adverse effects due to fear that this may decrease adherence.

Regarding adverse effects, emotional blunting and diarrhoea were greater concerns for patients. Doctors were more likely to discontinue lithium for fear of intoxications. Yet, lithium intoxications are relatively rare events, which can be safely managed in most cases [29]. Doctors were also more likely to stop lithium due to decreases in glomerular function. But even in many patients with a decrease in glomerular function, lithium can be continued, since the risk of suicide and recurrence of an acute manic or depressive episode tends to outweigh the risk of end-stage renal disease [1, 9, 10, 30, 31]. In any case, it has been suggested that renal function declines irrespective of lithium discontinuation – albeit slowly and after decades of treatment [16]. If this suggestion was confirmed, lithium discontinuation would be of no benefit at all.

### Length of time adverse effects are coped with

Our results suggest that some patients tried to cope with diarrhoea and polyuria over long periods of time, before they discontinued. As such adverse effects are perceived as stigmatising, patients may not bring them up. The same applies for side effects concerning sexual dysfunction, which in our study, only very few patients reported. Doctors may underestimate the personal significance of such adverse effects. The impact of adverse effects on quality of life of patients treated with lithium has not been studied [8]. Particularly polyuria and diabetes insipidus can become disabling, making patients virtually house-bound in extreme cases. Schou et al. considered nausea an early adverse event that became less common after long-term lithium use [32]. But our findings suggest that waiting for nausea to pass is not an option for most patients. Tremor is another adverse effect thought to improve over time [8]. However, our study does not confirm this assumption. Patients stayed on lithium for an average of four years before they discontinued lithium due to tremor.

### Strengths

Register studies have access to large sample sizes with little detailed clinical information. Conversely, clinical studies have access to detailed clinical information, but samples sizes tend to be small. In our study, we combined the best of both worlds, a large sample size and detailed clinical information. Thus, we established lithium treatment status, not only by prescription but also by lithium serum levels and information recorded in the medical notes. We were also able to validate diagnoses from the case records and distinguish between the various types of BPAD. All reasons for lithium were assessed by two separate reviewers to minimize observer bias. Our long follow-up time allowed to take account both, adverse effects occurring early and late during lithium treatment. A further strength was the high rate of consent for participation into the study. As age and sex distribution of consenting or not consenting patients was similar, the likelihood of selection bias was low.

### Limitations

Our study was retrospective and observational in nature. Hence, the quality of our data depended on the quality of the information recorded in the medical notes. Doctors may record selectively problems they find most relevant. This could lead to an underestimation of patients’ concerns. Besides, most patients have contact with more than one health professional, each of whom may focus on different issues.

Compared to retrospective observational studies, prospective studies run a higher risk that the very act of observation modifies the outcome under study. As described above, we took extensive measures to ensure that the data abstracted from the clinical notes was correct. Lithium discontinuation can be difficult to define in terms of time frames. Some patients may discontinue lithium nearly immediately after initiation. Others may have longer periods of lithium interruption due to physical illness, but finally resume treatment. Therefore, we considered lithium to be truly discontinued, when there was a stated intention to discontinue for good. Some patients had episodes of lithium treatment before our observation period began. Thus, it was possible to misclassify adverse effects as occurring early, when in fact they occurred late. For avoiding such misclassification, we needed to avoid missing such prior episodes of lithium discontinuation. For this analysis, we only included a subset of patients. For this subset, we could establish that patients had not had any episodes of lithium treatment that could potentially have gone unnoticed.

## Conclusions

With the mortality gap between individuals with bipolar affective disorder and the general population widening [33], we need to understand why patients and doctors discontinue lithium. It is important that patients who may benefit from lithium can continue to take it, striking the right balance between benefits and risks [9, 10]. Particularly men may require proactive follow-up since they may be more likely not to consult with their doctor before discontinuing treatment. Understanding the reasons why patients and doctors discontinue lithium can assist us to develop strategies to improve adherence. In this context, management of adverse effects plays a major role. Our results show that regular monitoring of laboratory parameters is necessary but not sufficient to manage adverse effects. Regular blood tests are no substitute for regular personal follow-up. Adverse effects such as diarrhoea and polyuria substantially impair quality of life, which must not be ignored. Unnecessary termination of treatment could possibly be avoided if patient received comprehensive information about their pharmacological treatment at an appropriate time. Being responsive to patients’ concerns and needs may substantially improve treatment adherence [34] Shared decision making (SDM), which has already been tested in primary care and psychiatric settings, may be a way forward [18, 19]. Finally, close collaboration between psychiatric and internal medicine/nephrology service is imperative to minimize discontinuation of lithium in patients who have capacity to benefit.

## Abbreviations

ANOVA: Analysis of variance
BALANCE trial: Bipolar Affective Disorder: Lithium/ Anticonvulsant Evaluation trial
BPAD: Bipolar affective disorder
i.e: id est
Li: Lithium
LISIE study: Lithium Effects and Side Effects study
MCH: Melanin concentrating hormone
n: Number
Nephrogenic DI: Nephrogenic diabetes insipidus
NICE: the UK National Institute for Clinical Excellence
SD: Standard deviation
SDM: Shared decision-making
SZD: Schizoaffective disorder
