# Correction to: Reasons for lithium discontinuation in men and women with bipolar disorder: a retrospective cohort study

**DOI:** 10.1186/s12888-018-1895-4

**Published:** 2018-10-03

**Authors:** Louise Öhlund, Michael Ott, Sofia Oja, Malin Bergqvist, Robert Lundqvist, Mikael Sandlund, Ellinor Salander Renberg, Ursula Werneke

**Affiliations:** 1 Sunderby Research Unit, Department of Clinical Sciences, Division of Psychiatry, Umeå University, Umeå, Sweden; 2 Department of Public Health and Clinical Medicine, Division of Medicine, Umeå University, Umeå, Sweden; 3 Department of Psychiatry, Sunderby Hospital, Luleå, Sweden; 4 Department of Psychiatry, Piteå Älvdals Hospital, Piteå, Sweden; 5 Research and Innovation Unit, Luleå, Norrbotten Region Sweden; 6 Department of Clinical Science, Division of Psychiatry, Umeå University, Umeå, Sweden; 7 Sunderby Hospital – Psychiatry, 97180 Luleå, Sweden

## Correction

Following publication of the original article [1], the authors reported an error in Fig. 1 and Table 1, concerning the number of female participants. The correct number is 283, instead of 238 that was originally published.

## References

[1] Öhlund L et al. (2018) Reasons for lithium discontinuation in men and women with bipolar disorder: a retrospective cohort study. BMC psychiatry 18:37. 10.1186/s12888-018-1622-1. 10.1186/s12888-018-1622-1 PMC5804058 29415689

